# Exogenous surfactant therapy in 2013: what is next? who, when and how should we treat newborn infants in the future?

**DOI:** 10.1186/1471-2431-13-165

**Published:** 2013-10-10

**Authors:** Emmanuel Lopez, Géraldine Gascoin, Cyril Flamant, Mona Merhi, Pierre Tourneux, Olivier Baud

**Affiliations:** 1 Service de Médecine Néonatale de Port-Royal Groupe Hospitalier Cochin-Broca-Hôtel Dieu, APHP, Paris, France; 2 Réanimation et Médecine Néonatales, CHU d’Angers, Angers, France; 3 Département de pédecine néonatale, CHU de Nantes, Nantes, France; 4 Réanimation Néonatale, CH Sud Francilien, Corbeil-Essonnes, France; 5 Médecine Néonatale, CHU d’Amiens, Amiens, France; 6 Réanimation et Pédiatrie Néonatales, Groupe Hospitalier Robert Debré, APHP, 48 Bd Sérurier, Paris, 75019, France

**Keywords:** Surfactant, Neonate, Respiratory distress, Developing lung, Critical care, Review

## Abstract

**Background:**

Surfactant therapy is one of the few treatments that have dramatically changed clinical practice in neonatology. In addition to respiratory distress syndrome (RDS), surfactant deficiency is observed in many other clinical situations in term and preterm infants, raising several questions regarding the use of surfactant therapy.

**Objectives:**

This review focuses on several points of interest, including some controversial or confusing topics being faced by clinicians together with emerging or innovative concepts and techniques, according to the state of the art and the published literature as of 2013. Surfactant therapy has primarily focused on RDS in the preterm newborn. However, whether this treatment would be of benefit to a more heterogeneous population of infants with lung diseases other than RDS needs to be determined. Early trials have highlighted the benefits of prophylactic surfactant administration to newborns judged to be at risk of developing RDS. In preterm newborns that have undergone prenatal lung maturation with steroids and early treatment with continuous positive airway pressure (CPAP), the criteria for surfactant administration, including the optimal time and the severity of RDS, are still under discussion. Tracheal intubation is no longer systematically done for surfactant administration to newborns. Alternative modes of surfactant administration, including minimally-invasive and aerosolized delivery, could thus allow this treatment to be used in cases of RDS in unstable preterm newborns, in whom the tracheal intubation procedure still poses an ethical and medical challenge.

**Conclusion:**

The optimization of the uses and methods of surfactant administration will be one of the most important challenges in neonatal intensive care in the years to come.

## 

Since the first successful studty by G. Enhoring and B. Robertson in 1972 demonstrating the effectiveness of natural lung surfactant administration in an immature rabbit model of respiratory distress syndrome (RDS) [1], many clinical studies have been carried out using synthetic or natural surfactant. Surfactant therapy is one of the few treatments that decreases overall mortality in preterm newborns with RDS, and has significantly changed clinical practice in neonatology. However, surfactant deficiency is also observed in many clinical situations other than RDS in term and preterm infants. This review focuses on the most controversial and confusing topics being faced by clinicians today, and emerging or innovative concepts and techniques regarding the use of surfactant therapy in respiratory management.

A systematic PubMed search up to January 2013 was undertaken to identify manuscripts addressing the following three specific questions:

1. Which infants should we treat with exogenous surfactant therapy?

2. When should preterm infants with RDS be treated with exogenous surfactant?

3. How should preterm infants with RDS be treated with exogenous surfactant?

## Which infants should we treat with exogenous surfactant therapy?

### Surfactant therapy for primary surfactant deficiency

#### Surfactant therapy for RDS in the preterm newborn

Surfactant synthesis starts early in fetal life and increases with gestational age. Over the last 10 years, meta-analyses have confirmed that exogenous surfactant treatment decreases overall morbidity and mortality in preterm newborns with RDS [2,3]. Both animal and human studies have demonstrated that early administration of surfactant is more effective than later rescue surfactant treatment because of better surfactant distribution and avoidance of ventilator-induced lung injury [4,5]. As of today, the questions that remain concerning surfactant therapy in preterm infants with RDS revolve around the identification of infants requiring surfactant, and the delivery method and dosage of surfactant administration. Indeed, emergency tracheal intubation in the delivery room for prophylactic or early surfactant administration raises ethical issues regarding pain management and the side effects induced by the procedure [6-8]. Other aspects of surfactant delivery, including the volume of surfactant administered, the rapidity of administration, drug viscosity and delivery rate, are also of interest. Finally, potential methods for the selection of infants with surfactant deficiency despite antenatal exposure to steroids include the stable microbubble test [9] and the click test, leading to earlier administration and reduced surfactant use [10].

#### Exogenous surfactant therapy for newborns of diabetic mothers

Epidemiological studies have shown that the risk of RDS is 5.6 times greater in newborn infants of diabetic mothers than in infants of non-diabetic mothers [11]. Although the strict management of maternal diabetes has reduced the incidence of RDS in very preterm infants of mothers with pregestational and gestational diabetes mellitus, pathophysiological data suggest that lung maturation is delayed in this population. In addition, although some studies show normal levels of disaturated phosphatidylcholine (DSPC), the main component of surfactant, in the amniotic fluid of diabetic pregnant women [12], others have revealed a decrease in DSPC levels in these pregnancies [13]. Even though these epidemiological and pathophysiological data suggest that the use of surfactant therapy would be beneficial in newborns born to diabetic mothers, no prospective study has as yet been performed in this population.

#### Newborns with genetic mutations in surfactant proteins

Lung diseases associated with surfactant metabolism dysfunctions represent a heterogeneous group of rare disorders [14], usually with poor prognosis and weak or transient effects of mechanical ventilation or exogenous surfactant therapy [15]. These conditions are rarely known before birth unless there has been a previously affected infant. The inherited deficiency of pulmonary surfactant B protein (SP-B) was first described in term newborns with RDS in 1993 [16]. Since then, other genetic mutations in surfactant proteins have been described, of which some induce RDS in newborns within the first few days of life while others result in lung diseases in older infants. These include mutations of the surfactant protein C (SP-C) gene [17], mutations in proteins required for surfactant synthesis, such as the ATP-binding cassette transporter, subfamily A, member 3 (ABCA3) [18] or the NK2 homeobox protein NKX2-1, a critical regulator of the transcription of SP-B and SP-C [19]. Steroids, hydroxychloroquine and azithromycin have been proposed in older patients, but little information is available to assess the benefit/risk ratio of these treatments.

### Surfactant therapy for secondary surfactant deficiency

Various clinical situations such as pulmonary haemorrhage, meconium aspiration syndrome (MAS), pulmonary infection and atelectasis have been shown to liberate inflammatory mediators that damage type II pneumocytes and inactivate surfactant [20,21]. Surfactant replacement therapy could thus be useful as a supporting treatment in this population of newborns with secondary or transient surfactant deficiency.

#### Surfactant therapy in term and near-term newborns with acute respiratory distress syndrome (ARDS)

The incidence of ARDS requiring mechanical ventilation in term and near-term newborns is 7.2/1000 live births, and 30% of newborns requiring mechanical ventilation in the neonatal intensive care unit (NICU) are low birth-weight infants [22]. The incidence of ARDS decreases from 10.5% (390/3700) for infants born at 34 weeks of gestation to 0.3% (140/41,764) at 38 weeks [23]. The incidence of respiratory morbidity is significantly higher in newborns delivered by caesarean section before the onset of labour (35.5/1000) than in those delivered by caesarean section during labour (12.2/1000) or in vaginal births (5.3/1000) [24]. Even among deliveries by caesarean section before the onset of labour, a significant reduction in the incidence of ARDS could be obtained if elective caesarean section is performed after the 39^th^ week of gestation [24,25]. Even if the overall incidence of ARDS seems low in term and near-term newborns, these still constitute a high-risk population with significant neonatal mortality and morbidity including air leaks, severe hypoxaemia, persistent pulmonary hypertension and bronchopulmonary dysplasia [26]. The mechanisms leading to ARDS in term or near-term newborns involve delayed lung liquid clearance and insufficient surfactant production. Similarly, term infants with transient tachypnea of the newborn have low lamellar body counts associated with decreased surfactant function, suggesting that prolonged disease is associated with surfactant abnormalities [27].

#### Surfactant therapy for newborns with pulmonary haemorrhage

Experimental data suggest that the molecular components involved in pulmonary haemorrhage can biophysically inactivate endogenous lung surfactant, whereas exogenous surfactant replacement is capable of reversing this process even in the continued presence of inhibitor molecules [28,29]. In two clinical studies, whose control groups were not comparable, the mean oxygenation index improved in preterm and term infants who received surfactant following clinically significant pulmonary haemorrhage, with no deterioration in the condition of any patient [30,31]. Case reports have also noted the successful use of surfactant treatment after idiopathic [32] or iatrogenic pulmonary haemorrhage [33]. However, a recent Cochrane meta-analysis found no any randomized or quasi-randomized trials evaluating the effects of surfactant in pulmonary haemorrhage in neonates [34], suggesting the need for such trials.

#### Surfactant therapy in meconium aspiration syndrome (MAS)

MAS is characterized by the early onset of respiratory distress in meconium-stained infants, resulting in high pulmonary morbidity and mortality [35,36]. The pathophysiology of MAS includes airway obstruction [37,38], alveolar inflammation [39] and surfactant inhibition [40,41]. Over the last 10 years, cohort studies assessing the use of treatments such as High-Frequency Oscillatory Ventilation (HFOV) or inhaled Nitric Oxide (iNO) in MAS have not revealed any decrease in the duration of ventilation or oxygen therapy [42,43].

Surfactant treatment has been proposed in MAS, either as a bolus treatment or surfactant lavage. In one meta-analysis, bolus surfactant treatment for MAS decreased the need for extracorporeal membrane oxygenation (ECMO) (NNT=6), but had no statistically significant effect on mortality, duration of assisted ventilation, duration of supplemental oxygen, pneumothorax, pulmonary interstitial emphysema, air leaks, chronic lung disease, need for oxygen at discharge or intraventricular haemorrhage [44]. Surfactant lavage has been performed in several animal and human studies, with an optimal total lavage fluid volume of 15 to 30 mL/kg [35,45,46]. The surfactant was diluted in these studies in physiological saline to obtain a final phospholipid concentration of 5 mg/mL [47]. In a recent meta-analysis of surfactant lavage, Hahn et al. state that lung lavage with diluted surfactant may be beneficial to infants with MAS, but additional controlled clinical trials of lavage therapy should be conducted to confirm this effect, to refine the method of lavage, and to compare lavage with other approaches [48]. In a study of newborn lambs with respiratory failure and pulmonary hypertension induced by MAS, gas exchange and lung compliance were improved by lung lavage with dilute surfactant but not by bolus treatment [49]. Given these results, it is safe to conclude that surfactant treatment, either as a bolus or diluted for lung lavage, would decrease the need for ECMO in human newborns with MAS. Furthermore, in infants with MAS, if ECMO is not available, surfactant administration may reduce the severity of respiratory illness and decrease the number of infants with progressive respiratory failure requiring support with ECMO. Larger clinical trials are necessary to confirm that surfactant may be an effective treatment for the aspiration of several biological fluids in addition to meconium, including blood, vernix and amniotic fluid.

### Surfactant therapy for impaired lung alveolarization

Both congenital and acquired lung growth impairments result in a decrease in lung alveolarization, type II pneumocyte counts and surfactant production [50-52], suggesting a potential benefit from surfactant replacement therapy.

#### Exogenous surfactant therapy for congenital diaphragmatic hernia (CDH)

Newborns with CDH display pulmonary hypoplasia with persistent pulmonary hypertension (PPH), resulting in a high incidence of respiratory morbidity and mortality [53,54]. Animal models of CDH have revealed a deficient surfactant system [50,55,56]. In human studies, Boucherat et al. have shown that CDH does not impair storage in fetuses [57]. CDH lungs exhibit no trend towards a decrease in content or a delay in developmental changes for any of the surfactant components or surfactant maturation factors studied. Data from cohorts of newborns with a prenatal diagnosis of isolated CDH do not show any benefit associated with surfactant therapy [53]. However, surfactant phosphatidylcholine synthesis is decreased in newborns with CDH who require ECMO after birth [58]. A plausible explanation for the difference in surfactant synthesis is that CDH infants who require ECMO have more severe pulmonary hypoplasia compared to CDH infants who do not require ECMO. Systematic surfactant therapy can thus not be recommended for term newborns with a prenatal diagnosis of isolated CDH. Whether surfactant therapy is beneficial or not in preterm or late preterm newborns with CDH who require ECMO should be evaluated in randomized trials that also take into account the severity of the underlying lung hypoplasia and gestational age at delivery.

#### Late surfactant therapy for chronically ventilated preterm infants

In spite of early exogenous surfactant treatment, extremely low birth weight infants can develop persistent respiratory failure during the first weeks of life, leading to bronchopulmonary dysplasia (BPD) and alveolarization defects [52]. Surfactant proteins are involved in the pulmonary host defence and response to lung injury. The synthesis of surfactant proteins has been found to be decreased in animal models of BPD [59]. Preterm infants requiring chronic ventilation after 7 days of life also present dysfunctional surfactant proteins [60].

Studies evaluating the effects of surfactant administration in chronically ventilated preterm infants have demonstrated a short-term beneficial effect on the fraction of inspired oxygen (FiO_2_) and the respiratory distress severity score at 48 and 72 hours [61]. However, the sole study to evaluate the effect of late surfactant treatment on the incidence of BPD or mortality has reported trends toward lower morbidity/mortality only in infants who received high dose of lucinactant [62].

## When should preterm infants with rds be treated with exogenous surfactant?

The optimal timing (prophylactic or selective) for the administration of surfactant to preterm infants with RDS has been assessed by many studies, and discussed in recent reviews [63]. On the basis of these studies, various guidelines have been elaborated by national expert committees in accordance with current practice and conclusions drawn from recent large trials of CPAP.

### Prophylactic vs. selective surfactant treatment

Rojas-Reyes et al. [5] have carried out a meta-analysis comparing the effectiveness of prophylactic vs. selective exogenous surfactant administration in preventing morbidity and mortality in very preterm infants below 30–32 weeks gestational age (GA). Prophylactic administration decreases the incidence of pneumothorax, pulmonary interstitial emphysema, neonatal mortality and BPD or death to a greater extent than selective treatment. However, several limitations of this meta-analysis should be noted: (i) the range of gestational ages studied was large, (ii) the exogenous surfactant used was natural but was different in each study, and (iii) the timing of selective surfactant administration was very different among the studies, from 1 hour to 24 hours after birth. In addition, the beneficial effect of prophylactic surfactant on neonatal mortality or air-leak syndromes was only seen in infants not routinely subjected to CPAP. Finally, the increasing use of antenatal betamethasone in the current era could be an explanation for the lower impact of prophylactic surfactant.

### Early vs. late surfactant treatment

The benefits of early (< 2 hours) and delayed (> 2 hours) surfactant administration have been recently reviewed [4] in a meta-analysis of six randomized controlled trials (RCTs), consisting of two trials with synthetic (Exosurf Neonatal) and four using animal-derived surfactant preparations [64-69]. According to this meta-analysis, early selective surfactant administration to infants with RDS requiring assisted ventilation leads to a decreased risk of acute pulmonary injury (decreased risk of pneumothorax and pulmonary interstitial emphysema) and a decreased risk of neonatal mortality and chronic lung disease, compared to delaying treatment of such infants until they develop worsening RDS.

More recently, two new RCTs have demonstrated that routine early surfactant administration within 2 hours of life:

–reduces the need for mechanical ventilation in the first week of life among preterm infants with RDS on nasal CPAP, born between 28 and 32 weeks GA [70],

–decreases intra-ventricular haemorrhage (≥ grade III) and pneumothorax rates but does not have any effect on BPD when compared to delayed surfactant administration [71].

### National guidelines for exogenous surfactant administration

Table 1 summarizes national recommendations for surfactant prophylactic use. The British Association of Perinatal Medicine recommended in 1999 that very preterm infants, born before 32 weeks GA be treated with exogenous surfactant at birth only if they needed intubation, and that all very preterm infants below 29 GA be intubated for the administration of exogenous surfactant [72]. More recently, in 2008, the American Academy of Pediatrics Committee on the Fetus and Newborn has recommended using surfactant in infants with RDS as soon as possible after intubation, irrespective of exposure to antenatal steroids or gestational age. They have also recommended that prophylactic surfactant treatment be administered to extremely preterm infants (< 28 weeks GA) at high risk of RDS, especially infants who have not been exposed to antenatal steroids [73]. The Canadian Paediatric society has advocated that intubated infants with RDS receive exogenous surfactant therapy, and that infants at significant risk of RDS receive prophylactic surfactant treatment as soon as they are stable, within a few minutes of intubation [74]. The consensus guidelines developed by European experts in neonatology recommend prophylactic surfactant administration to all extremely preterm infants born at less than 26 weeks GA and to all preterm infants with RDS who require intubation for stabilization. In addition, they recommend that early rescue surfactant therapy be administrated to untreated preterm infants with RDS [75]. In a recent update of European consensus guidelines on the management of neonatal respiratory distress syndrome in preterm infants, the experts state that the best preparation, optimal dose and timing of surfactant administration at different gestational ages is not completely clear. In addition, the use of very early CPAP has altered the indications for prophylactic surfactant administration [76].

**Table 1:** International guidelines for RDS treatment

**Country (ref)**	**Year**	**Gestational age**	**Prophylactic use of surfactant**
**UK (72)**	**1999**	**< 29 weeks**	**Systematic**
		**< 32 weeks**	**If need for intubation at birth**
**Canada (74)**	**2005**	**< 26 weeks**	**Systematic**
		**26-27 weeks**	**If no antenatal steroids**
**US (73)**	**2008**	**< 28 weeks**	**Systematic**
**Europe (75–76)**	**2010-2013**	**< 26 weeks**	**Systematic**

A European survey conducted in 2011 has analysed the incorporation of guidelines for surfactant therapy into clinical practice in 173 NICUs across 21 European countries [77]. Only 39% of the NICUs used prophylactic treatment. Twenty-three % of preterm infants received their first surfactant dose within the first 15 minutes after birth, while 28% of them received it after 2 hours of life. A gestational age of less than 28 weeks and a birth weight of less than 1000 g were used as criteria for prophylactic treatment in most of NICUs. Eighty eight % used a median FiO_2_ of greater than 0.40 as the indication for rescue surfactant treatment, at a median time of 2 hour after birth.

### CPAP vs. intubation for exogenous surfactant infusion in the age of antenatal corticosteroids

There is increasing evidence to suggest that CPAP immediately after birth is a reasonable alternative to systematic intubation for surfactant administration to preterm infants. Recent trials on this topic are summarized in Table 2.

**Table 2:** Recent studies concerning CPAP and surfactant administration

**Study (ref)**	**Year**	**N**	**Population**	**Outcome**	**Groups**		
**COIN Trial (78)**	2008	610	25-28 wks		**CPAP**		**Systematic intubation**
				Death or BPD	33.9%		38.9%
				Intubation	46%		100%
				Selective surfactant	38%***		77%
				Pneumothorax	9%***		3%
**Rojas et al. (79)**	2009	279	27-31 wks		**CPAP**		**Intubation-early surfactant-extubation**
				Need for MV	39%*		26%
				Pneumothorax	9%		2%
				Rescue surfactant	26%**		12%
				BPD at 36 wks PMA	59%		49%
**CURPAP Trial (80)**	2010	208	25-28 wks		**CPAP Early selective Surfactant**		**Prophylactic surfactant**
				Need for MV	33%		31.4%
				Death at 36 wks PMA	10.7%		8.6%
				Pneumothorax	1%		6.7%
				BPD	11.7%		14.3%
**SUPPORT Trial (81)**	2010	1316	24-28 wks		**CPAP**		**Intubation-early surfactant use**
				Death or BPD	48.7%		54.1%
				Surfactant use	67.1%***		98.9%
				Air leak	6.8%		7.4%
**Dunn et al. (82)**	2011	648	26-29 wks		**CPAP Selective surfactant**	**Prophylactic surfactant**	**Intubation-early surfactant-extubation**
				Surfactant use	14.8%***	98.6%	98.2%
				Death or BPD	30.5%	36.5%	28.5%
				Pneumothorax	5.4%	4.8%	3.2%

BPD=bronchopulmonary dysplasia, PMA=postmenstrual age, MV=mechanical ventilation; wks: weeks of gestation.

*:p<0.05; **:p<0.01; ***:p<0.001.

Morley et al. [78] demonstrated, in the COIN trial, that early nasal CPAP did not reduce the rate of death or BPD, but the need for intubation and use of surfactant were halved (38% vs. 77%; p<0.001) in extremely preterm infants. This study suggests that starting respiratory support with CPAP did not adversely affect infants even if the incidence of pneumothorax was increased in the CPAP group when compared with the intubation group (9% vs. 3%; p<0.001). Rojas et al. [79] conducted a trial showing that an “intubation-very early surfactant administration-extubation” sequence improved outcome in very preterm infants by decreasing the need for mechanical ventilation and the incidence of air-leak syndromes, when compared with CPAP therapy alone. In the CURPAP trial [80], prophylactic surfactant administration to very preterm infants was not superior to CPAP or early selective surfactant treatment in reducing the need for mechanical ventilation, pneumothorax, BPD or mortality.

In the SUPPORT trial [81], the authors randomized 1316 infants born at 24–27 weeks GA into two groups: a CPAP group and a surfactant group (intubation and prophylactic surfactant administration). The primary outcome (death or BPD at 36 weeks) did not differ significantly between the two groups. In addition, a high proportion (46%) of infants assigned to the “initial CPAP” group still ended up being intubated and receiving surfactant. Dunn et al. [82] compared 3 strategies for initial respiratory management in infants born between 26 and 29 weeks GA: 1) prophylactic surfactant administration followed by mechanical ventilation, 2) intubation-early surfactant use-extubation and 3) initial CPAP with selective surfactant treatment. The incidence of death or BPD at 36 weeks was similar in the 3 groups. No significant differences were found at 18 to 22 months of corrected age with regard to the composite outcome of death or neurodevelopmental impairment between extremely premature infants randomly assigned to early CPAP or early surfactant administration and to a lower or higher target range of oxygen saturation [83]. These controlled studies demonstrate that early CPAP use safely reduces the number of infants intubated and treated with surfactant compared to prophylactic surfactant administration or the intubation-surfactant-extubation approach. Unfortunately, neither the SUPPORT nor the COIN trial helps to identify infants at birth who, if initially ventilated using CPAP, will subsequently require intubation and ventilation.

### Prophylactic vs. selective surfactant administration in the age of antenatal corticosteroids and CPAP

Rojas-Reyes et al. [5] have recently updated a meta-analysis comparing the effects of prophylactic surfactant with selective surfactant treatment. The benefits of the prophylactic surfactant strategy demonstrated by the first meta-analysis are not confirmed by this updated meta-analysis. Recent large trials that reflect the current increase in antenatal corticosteroid administration and routine post-delivery stabilization on CPAP also do not support these benefits.

In summary, early selective surfactant treatment in preterm infants with RDS is more effective than delayed selective surfactant use in reducing neonatal mortality, pneumothorax, and BPD at 36 weeks. Prophylactic treatment also decreases the incidence of pneumothorax, pulmonary interstitial emphysema, BPD and neonatal mortality when compared with delayed selective treatment. However, prophylactic surfactant treatment is not superior to initial respiratory support with CPAP followed by selective surfactant treatment later on in reducing the need for mechanical ventilation, pneumothorax and BPD or mortality in the era of antenatal corticosteroids and CPAP.

## How should preterm infants with rds be treated with exogenous surfactant: InSurE or MIST?

### The “InSurE” strategy

The InSurE approach is a strategy in which surfactant is administered during brief intubation followed by immediate extubation and nasal respiratory support. This strategy has been shown in earlier RCTs to halve the need for subsequent mechanical ventilation. More recently, Bhandari et al. have demonstrated that the InSurE strategy is associated with a significantly lower incidence of BPD or death (20% vs. 52%; p=0.03) [84].

A Cochrane review updated in 2007 has compared InSurE with later selective surfactant use. The InSurE strategy is associated with a significantly lower incidence of mechanical ventilation and a trend towards a decrease in BPD and air-leak syndromes [85]. Rojas et al. evaluated very early surfactant administration with early CPAP in very preterm infants [79]. In the InSurE group, the need for mechanical ventilation was significantly lower and air-leak syndromes were less frequent, but the decrease in the incidence of BPD did not reach statistical significance. Dani et al. have identified the clinical characteristics that could predict the success or failure of InSurE in infants [86]. Gestational age, birth weight and the need for oxygen are independent risk factors for InSurE failure in infants. Kirsten et al. have also reported in a recent observational study that early neonatal outcome in extremely immature infants may be improved by the administration of antenatal steroids as well as the InSurE strategy [87].

Taken together, these various studies demonstrate that InSurE is a safe and effective method that reduces the need for mechanical ventilation, the duration of respiratory support and the need for surfactant replacement in preterm infants with RDS. It may also reduce the rate of BPD and air-leak syndromes, but limited data are available regarding cerebral oxygenation and the potential risk of brain damage. This strategy needs to be individually tailored in accordance with the infant’s maturity and general clinical condition, the use of antenatal steroid treatment, the severity of RDS and certain practical considerations affecting the NICU.

### Emerging techniques for surfactant administration

Considering the risks associated with the use of an endotracheal tube (usually associated with premedication that contributes to a delay in extubation), Minimally-Invasive Surfactant Therapy (MIST) and Non-Invasive Surfactant Therapy (NIST) are emerging methods for surfactant administration without the need for positive-pressure mechanical ventilation (Table 3) [88].

**Table 3:** Emerging approaches to surfactant administration

**Emerging approaches**		**Advantages**	**Disadvantages**
**MIST**	Nasopharyngeal instillation	Painless	Not well evaluated
			Loss of surfactant
	Laryngeal mask	Supraglottic device	Painful
	Feeding catheter	Endotracheal administration Under nasal CPAP	Magill forceps
			Laryngoscopy
			Painful and traumatic?
	Vascular catheter	Endotracheal administration	Laryngoscopy
		Under nasal CPAP	Painful and traumatic
		Easy to use (rigid catheter)	
**NIST**	Aerosolization	Painless	Technically challenging

MIST: Minimally-Invasive Surfactant Therapy.

NIST: Non-Invasive Surfactant Therapy.

#### Should surfactant always be administered through an endotracheal tube?

### Intrapartum pharyngeal instillation

An initial multicenter trial tested surfactant administration to the posterior pharynx immediately following birth, before the first inspiration of air [89]. In this trial, the solution was not administered under CPAP and many of the subjects also received surfactant through an endotracheal tube. More recently, 23 neonates born between 27 and 30 weeks GA were treated by surfactant instilled into the nasopharynx before the delivery of the shoulders. Thirteen of 15 babies delivered vaginally were weaned quickly to room air and required no further surfactant or tracheal intubation for RDS. Evidence from animal and observational human studies suggests that the pharyngeal instillation of surfactant before the first breath is potentially safe, feasible and effective. Well-designed trials are still needed to confirm this [90].

### Laryngeal mask

The laryngeal mask airway (LMA) is a supraglottic device used to administer non-invasive pressure ventilation to adult, paediatric and neonatal patients. Trevisanuto et al. used a laryngeal mask to deliver surfactant to 8 preterm and near-term infants with RDS and a birth weight > 800 g [91], leading to an increased mean arterial/alveolar oxygen tension ratio (a/APO_2_) without complications. The Cochrane review cited above [90] reported no study of prophylactic surfactant administration using an LMA. The Cochrane experts concluded that there was some evidence that selective surfactant administration through an LMA to preterm infants > 1200 g with established RDS reduced oxygen requirement in the short term, although the LMA technique needed to be further evaluated in clinical trials, including those dedicated to investigating the size of the LMA used according to gestational age.

### Feeding and vascular catheter

An alternative route for surfactant administration in spontaneously breathing preterm infants on CPAP consists of introducing surfactant via a thin endotracheal catheter under laryngoscopy. The tube is inserted only for the few minutes necessary to administer surfactant. The first cases using this technique were reported by Verder et al. [92]. A recent multicentre RCT including very preterm infants has compared a standard group (CPAP, rescue intubation and surfactant treatment if needed) with an intervention group (surfactant via thin catheter) [93]. According to this non-blinded trial, the new method reduces the need for subsequent mechanical ventilation on day 2 or 3 after birth, and the duration of mechanical ventilation as well as the need for oxygen therapy at 28 days. However, the risk of death or BPD at 36 weeks does not differ between the two groups, and the trial has prompted a few methodological concerns. These findings may be of interest for infants that have received antenatal steroids.

The use of a more stable vascular catheter (the Hobart method) for surfactant administration allows easier placement without the use of Magill forceps [94]. A preliminary evaluation of this new technique in preterm infants showed that surfactant was successfully administered and CPAP quickly re-established, with decreasing FiO_2_. Transient coughing (32%) and bradycardia (44%) were noted, and 44% of patients received positive-pressure inflation. Multicentre RCTs of surfactant administration via catheterization in preterm infants on CPAP are ongoing (the OPTIMIST-A and -B trials). In active preterm infants, the placement of a catheter without sedation may be difficult and potentially traumatic. Guidelines for premedication are therefore needed to minimize this risk.

#### Non-Invasive Surfactant Therapy (NIST): aerosolized surfactant

Within a few years of Patrick Kennedy’s death from RDS in 1963, the first two trials reporting the use of synthetic surfactants were published [95,96]. Both used nebulized dipalmitoyl-phosphatidylcholine, with no discernible beneficial effects. Since then, several pilot trials have been published, but it has not been possible to clearly demonstrate whether surfactant can be successfully administered via nebulization [97]. To be effective, the surfactant would have to be unaltered by the aerosolization process: i.e. capable of being delivered to distal portions of the lung, reaggregating, and maintaining its biological activity. Several animal studies have directly compared aerosolization to tracheal instillation, and reported that aerosolization can be of equal or greater effectiveness [98], whereas others have found that instillation is more effective than aerosolization [99]. An example illustrating the need for further clarity on this issue is the study by Lewis et al. [100]. In sheep, exogenous bovine lung extract surfactant was effective when instilled but ineffective when aerosolized, whereas beractant in the same animal model was more effective when aerosolized than when instilled. Aerosol delivery to the alveoli in normal lungs is maximal in the particle-size range 0.5-2.0 μm, and it is possible to generate a stable surfactant aerosol with that particle-size range from aqueous or powdered surfactant [101]. Currently, 3 types of nebulizers are available: jet nebulizers, ultrasound nebulizers and vibrating-membrane nebulizers. If aerosol delivery technology for lung surfactant could be perfected, this would clearly be a conceptually attractive alternative to instillation for clinical application. Indeed, aerosol delivery might avoid the transient endotracheal tube obstruction and resultant hypoxia and hypotension seen with bolus instillation. However, delivering aerosolized surfactant in sufficient quantities and achieving its uniform distribution throughout the alveoli of injured lungs in ventilated units is a key challenge. Whether surfactant aerosolization can be accomplished in a sufficiently effective and efficient manner to replace instillation requires further direct comparisons in animals and subsequently in human trials. A recent clinical study using aerosolized lucinactant has demonstrated the feasibility and safety of delivering this synthetic-peptide-containing surfactant to newborns with early signs of RDS [102]. Nebulized surfactant is potentially a therapeutic option to avoid the severe volutraumatic and barotraumatic effects of mechanical ventilation. However, several issues regarding cost-effectiveness, the development of nebulizer devices capable of its administration, and dosing strategies remain unresolved [103]. An open-label randomized controlled pilot study to evaluate the safety and efficacy of aerosolized porcine surfactant in the first hour of life in preterm infants with RDS (CureNeb) is ongoing in Australia. The primary outcomes evaluated are the duration of mechanical ventilation and the need for intubation.

In conclusion, surfactant administration via an endotracheal tube remains the gold standard for surfactant administration in intubated infants. However, CPAP, as the primary respiratory support technique in infants with RDS, has been shown to be at least as effective as mechanical ventilation, and newly emerging techniques for surfactant administration in non-ventilated infants are currently under investigation. The optimization of a less- or non-invasive method of surfactant administration will be one of the most important challenges in the field of surfactant therapy for RDS in the coming years.

## Potential adverse effects and safety of exogenous surfactant therapy

The short-term risks of surfactant administration include bradycardia and hypoxemia during instillation, as well as blockage of the endotracheal tube [104]. The relative risk of pulmonary haemorrhage following surfactant therapy has been reported at 1.47 (95% CI: 1.05 to 2.07) in trials [105], but this adverse event is rarely reported in many of the RCTs of surfactant administration. Moreover, a recent meta-analysis that included 4 trials did not show any significant difference in the risk of pulmonary haemorrhage between prophylactic vs. selective administration of surfactant [5]. Due to the very rapid improvement in gas exchange in surfactant-treated infants, overdistension and hyperventilation with low partial pressure of carbon dioxide (PCO_2_) can occur. No other adverse clinical outcome has been shown to be increased by surfactant replacement. However, emerging techniques for surfactant administration may give rise to new and unexpected adverse events that must be taken into account in current and future studies.

To date, there is no evidence that there are any immunological changes of clinical concern due to bovine or porcine sources of proteins contained in natural surfactants [106].

## Competing interests

The authors declare that they have no competing interests.

## Authors’ contributions

EL wrote the “WHEN SHOULD PRETERM INFANTS WITH RDS BE TREATED WITH EXOGENOUS SURFACTANT?” section. PT wrote the “WHICH INFANTS SHOULD WE TREAT WITH EXOGENOUS SURFACTANT THERAPY?” section. GG, CF and MM wrote the “HOW SHOULD PRETERM INFANTS WITH RDS BE TREATED WITH EXOGENOUS SURFACTANT: INSURE OR MIST?” section. OB conceived of the organization of the review, participated in its coordination and helped to draft the manuscript. All authors from the “French Young Neonatologist Club” read and approved the final manuscript.

## Pre-publication history

The pre-publication history for this paper can be accessed here:

http://www.biomedcentral.com/1471-2431/13/165/prepub
